# (3R*,5′S*)-6,7-Dimeth­oxy-3-(4′-meth­oxy-6′-methyl-5′,6′,7′,8′-tetra­hydro-1,3-dioxolo[4,5-*g*]isoquinolin-5′-yl)isobenzofuran-1(3*H*)-one (racemic α-noscapine)

**DOI:** 10.1107/S1600536810003958

**Published:** 2010-02-10

**Authors:** Jan von Langermann, Heike Lorenz, Oliver Boehm, Anke Flemming, Arne Bernsdorf, Martin Köckerling, Dieter Schinzer, Andreas Seidel-Morgenstern

**Affiliations:** a Max-Planck-Institute for Dynamics of Complex Technical Systems, Sandtorstrasse 1, 39106 Magdeburg, Germany; b MOLISA GmbH, Brenneckestrasse 20, 39118 Magdeburg, Germany; c University of Rostock, Department of Chemistry, Devision of Inorganic Chemistry, Albert-Einstein-Strasse 3a, D-18059 Rostock, Germany

## Abstract

In the racemic title compound, C_22_H_23_NO_7_, the dihedral angle between the fused ring systems is 51.87 (6)°. Two of the meth­oxy groups are disordered over two orientations in 0.688 (5):0.312 (5) and 0.672 (15):0.328 (15) ratios. In the crystal, weak C—H⋯O inter­actions link the mol­ecules.

## Related literature

For the anti­tussive properties of *S*,*R*-noscapine [(−)-narcotin], a main alkaloid of the opium poppy, see: Bergmann & Stolzer (1956 ▶). For the biological activity of noscapine and related compounds, see: Aneja *et al.* (2006 ▶, 2007 ▶); Mahmoudian *et al.* (2009 ▶); Dahlstrom *et al.* (1982 ▶); Anderson *et al.* (2005 ▶). For the crystal structure of the naturally occurring chiral mol­ecule, see: Seetharaman *et al.* (1995 ▶).
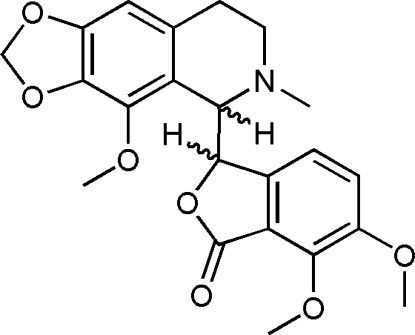


## Experimental

### 

#### Crystal data

C_22_H_23_NO_7_
*M* *_r_* = 413.41
Monoclinic, 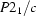

*a* = 15.5242 (8) Å
*b* = 9.3581 (5) Å
*c* = 13.2801 (7) Å
β = 95.781 (2)°
*V* = 1919.48 (17) Å^3^
*Z* = 4
Mo *K*α radiation
μ = 0.11 mm^−1^
*T* = 173 K
0.59 × 0.36 × 0.11 mm

#### Data collection

Bruker SMART CCD diffractometer
Absorption correction: multi-scan (*SADABS*; Bruker, 2007 ▶) *T*_min_ = 0.939, *T*_max_ = 0.988
14003 measured reflections
3864 independent reflections
2989 reflections with *I* > 2σ(*I*)
*R*_int_ = 0.018

#### Refinement

*R*[*F* ^2^ > 2σ(*F* ^2^)] = 0.057
*wR*(*F* ^2^) = 0.169
*S* = 1.12
3864 reflections
310 parameters
H-atom parameters constrained
Δρ_max_ = 0.56 e Å^−3^
Δρ_min_ = −0.71 e Å^−3^

### 

Data collection: *SMART* (Bruker, 2007 ▶); cell refinement: *SAINT* (Bruker, 2007 ▶); data reduction: *SAINT*; program(s) used to solve structure: *SHELXS97* (Sheldrick, 2008 ▶); program(s) used to refine structure: *SHELXL97* (Sheldrick, 2008 ▶); molecular graphics: *SHELXTL* (Sheldrick, 2008 ▶); software used to prepare material for publication: *SHELXTL*.

## Supplementary Material

Crystal structure: contains datablocks I, global. DOI: 10.1107/S1600536810003958/hb5290sup1.cif

Structure factors: contains datablocks I. DOI: 10.1107/S1600536810003958/hb5290Isup2.hkl

Additional supplementary materials:  crystallographic information; 3D view; checkCIF report

## Figures and Tables

**Table 1:** Hydrogen-bond geometry (Å, °)

*D*—H⋯*A*	*D*—H	H⋯*A*	*D*⋯*A*	*D*—H⋯*A*
C6—H6*A*⋯O1^i^	1.00	2.54	3.533 (3)	172
C13—H13*A*⋯O2^ii^	1.00	2.44	3.317 (3)	146
C18—H18*A*⋯O5^iii^	0.95	2.34	3.120 (3)	140

Symmetry codes: (i) 
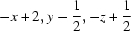
; (ii) 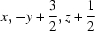; (iii) 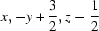.

**Fig. 1.**
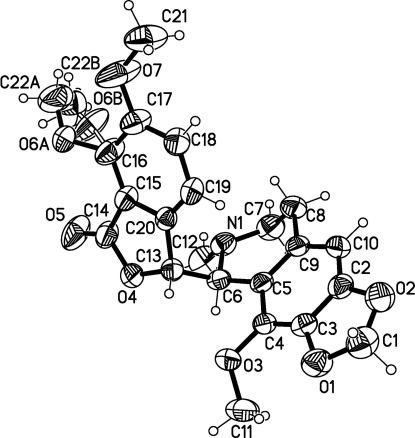
The molecular structure of (I) with displacement ellipsoids shown at the 50% probability level. The O6—C22H3 methoxy group is disordered on two positions.

**Fig. 2.**
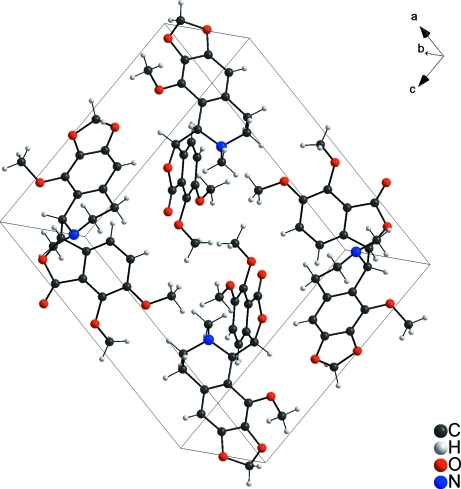
Partial packing diagram of (I).

## supplementary crystallographic information

### Comment

The antitussive properties of S,*R*-noscapine [(-)-narcotin], a main alkaloid of opium poppy, were investigated for several decades (e.g. Bergmann *et al.*, 1956). Also anti-cancer properties were recently discussed.  Unfortunately, the main production source of this compound is still the illegal crop growing. Therefore, for a drug-independent noscapine-source total synthesis is required, which yield in racemic alpha-noscapine (racemic mixture of S,R- and *R*,S-noscapine). This compound may be used as an intermediate to obtain S,*R*-noscapine by separation procedures. Its synthesis will be reported later.

For the biological activity of noscapine and related compounds, see: Aneja *et al.* (2007); Mahmoudian & Rahimi-Moghaddam (2009); Dahlstrom *et al.* (1982); Aneja *et al.* (2006); Anderson *et al.* (2005). For the crystal structure of the naturally occurring chiral molecule, see: Seetharaman & Rajan (1995).

### Refinement

The H atoms were geometrically placed (C—H = 0.95–1.00Å) and refined as riding with U_iso_(H) = 1.2U_eq_(C) or 1.5U_eq_(methyl C).  The deepest difference hole is -0.71 e/Å^3^ at x = 0.3614, y = 0.2316, z = 0.5208 (0.85 Å apart from C16).

### Crystal data

**Table:**

C_22_H_23_NO_7_	*F*(000) = 872
*M**_r_* = 413.41	*D*_x_ = 1.431 Mg m^−^^3^
Monoclinic, *P*2_1_/*c*	Melting point: 501.9 K
Hall symbol: -P 2ybc	Mo *K*α radiation, λ = 0.71073 Å
*a* = 15.5242 (8) Å	Cell parameters from 6930 reflections
*b* = 9.3581 (5) Å	θ = 2.5–29.9°
*c* = 13.2801 (7) Å	µ = 0.11 mm^−^^1^
β = 95.781 (2)°	*T* = 173 K
*V* = 1919.48 (17) Å^3^	Block, colourless
*Z* = 4	0.59 × 0.36 × 0.11 mm

### Data collection

**Table:**

Bruker SMART CCD diffractometer	3864 independent reflections
Radiation source: fine-focus sealed tube	2989 reflections with *I* > 2σ(*I*)
graphite	*R*_int_ = 0.018
ω scans	θ_max_ = 26.3°, θ_min_ = 3.4°
Absorption correction: multi-scan (*SADABS*; Bruker, 2007)	*h* = −18→19
*T*_min_ = 0.939, *T*_max_ = 0.988	*k* = −11→8
14003 measured reflections	*l* = −15→16

### Refinement

**Table:**

Refinement on *F*^2^	Secondary atom site location: difference Fourier map
Least-squares matrix: full	Hydrogen site location: inferred from neighbouring sites
*R*[*F*^2^ > 2σ(*F*^2^)] = 0.057	H-atom parameters constrained
*wR*(*F*^2^) = 0.169	*w* = 1/[σ^2^(*F*_o_^2^) + (0.0785*P*)^2^ + 1.390*P*] where *P* = (*F*_o_^2^ + 2*F*_c_^2^)/3
*S* = 1.12	(Δ/σ)_max_ = 0.001
3864 reflections	Δρ_max_ = 0.56 e Å^−^^3^
310 parameters	Δρ_min_ = −0.71 e Å^−^^3^
0 restraints	Extinction correction: *SHELXL97* (Sheldrick, 2008), Fc^*^=kFc[1+0.001xFc^2^λ^3^/sin(2θ)]^-1/4^
Primary atom site location: structure-invariant direct methods	Extinction coefficient: 0.0057 (18)

### Special details

**Table:**

Geometry. All e.s.d.'s (except the e.s.d. in the dihedral angle between two l.s. planes) are estimated using the full covariance matrix. The cell e.s.d.'s are taken into account individually in the estimation of e.s.d.'s in distances, angles and torsion angles; correlations between e.s.d.'s in cell parameters are only used when they are defined by crystal symmetry. An approximate (isotropic) treatment of cell e.s.d.'s is used for estimating e.s.d.'s involving l.s. planes.
Refinement. Refinement of *F*^2^ against ALL reflections. The weighted *R*-factor *wR* and goodness of fit *S* are based on *F*^2^, conventional *R*-factors *R* are based on *F*, with *F* set to zero for negative *F*^2^. The threshold expression of *F*^2^ > σ(*F*^2^) is used only for calculating *R*-factors(gt) *etc*. and is not relevant to the choice of reflections for refinement. *R*-factors based on *F*^2^ are statistically about twice as large as those based on *F*, and *R*- factors based on ALL data will be even larger.

### Fractional atomic coordinates and isotropic or equivalent isotropic displacement parameters (Å^2^)

**Table:**

	*x*	*y*	*z*	*U*_iso_*/*U*_eq_	Occ. (<1)
N1	0.73446 (12)	0.3640 (2)	0.27455 (14)	0.0344 (5)	
O1	1.04337 (10)	0.66824 (18)	0.09956 (12)	0.0386 (4)	
O2	0.95148 (11)	0.68699 (18)	−0.04776 (12)	0.0404 (4)	
O3	0.98826 (10)	0.53115 (19)	0.28872 (11)	0.0374 (4)	
O4	0.79757 (10)	0.49937 (17)	0.46248 (11)	0.0342 (4)	
O5	0.69655 (18)	0.5011 (2)	0.56862 (18)	0.0843 (9)	
C1	1.03115 (17)	0.7361 (3)	0.00278 (19)	0.0428 (6)	
H1A	1.0296	0.8412	0.0111	0.051*	
H1B	1.0796	0.7120	−0.0373	0.051*	
C2	0.90820 (15)	0.6242 (2)	0.02642 (16)	0.0324 (5)	
C3	0.96312 (14)	0.6128 (2)	0.11444 (17)	0.0305 (5)	
C4	0.93703 (14)	0.5473 (2)	0.19924 (16)	0.0287 (5)	
C5	0.85049 (13)	0.4988 (2)	0.19361 (15)	0.0284 (5)	
C6	0.81858 (13)	0.4371 (2)	0.28879 (16)	0.0293 (5)	
H6A	0.8627	0.3669	0.3183	0.035*	
C7	0.70645 (16)	0.3236 (3)	0.16978 (19)	0.0412 (6)	
H7A	0.6475	0.2824	0.1660	0.049*	
H7B	0.7459	0.2496	0.1473	0.049*	
C8	0.70633 (15)	0.4513 (3)	0.10059 (18)	0.0412 (6)	
H8A	0.6858	0.4225	0.0305	0.049*	
H8B	0.6664	0.5251	0.1224	0.049*	
C9	0.79610 (14)	0.5116 (2)	0.10352 (16)	0.0323 (5)	
C10	0.82522 (14)	0.5736 (2)	0.01770 (16)	0.0343 (5)	
H10A	0.7889	0.5805	−0.0441	0.041*	
C11A	1.07435 (17)	0.4888 (4)	0.2848 (2)	0.0634 (9)	
H11A	1.1034	0.4824	0.3537	0.095*	
H11B	1.1042	0.5592	0.2461	0.095*	
H11C	1.0757	0.3953	0.2518	0.095*	
C13	0.81365 (13)	0.5602 (2)	0.36560 (15)	0.0279 (5)	
H13A	0.8699	0.6132	0.3730	0.033*	
C14	0.72208 (18)	0.5464 (3)	0.4926 (2)	0.0451 (6)	
C15	0.68646 (15)	0.6537 (2)	0.41869 (19)	0.0379 (6)	
C16	0.61460 (19)	0.7398 (3)	0.4222 (3)	0.0668 (10)	
C17	0.59567 (16)	0.8363 (3)	0.3418 (2)	0.0511 (7)	
C18	0.64984 (15)	0.8462 (2)	0.26480 (17)	0.0358 (5)	
H18A	0.6367	0.9123	0.2111	0.043*	
C19	0.72308 (15)	0.7602 (2)	0.26562 (16)	0.0348 (5)	
H19A	0.7604	0.7682	0.2134	0.042*	
C20	0.74082 (13)	0.6636 (2)	0.34276 (15)	0.0266 (5)	
C12	0.73227 (19)	0.2382 (3)	0.3401 (2)	0.0483 (7)	
H12A	0.6754	0.1924	0.3286	0.073*	
H12B	0.7428	0.2677	0.4110	0.073*	
H12C	0.7772	0.1705	0.3244	0.073*	
O6A	0.57726 (16)	0.7471 (3)	0.51826 (17)	0.0462 (9)	0.688 (5)
C22A	0.4909 (3)	0.7090 (9)	0.5089 (4)	0.0661 (17)	0.688 (5)
H22A	0.4731	0.6860	0.5758	0.099*	0.688 (5)
H22B	0.4822	0.6252	0.4647	0.099*	0.688 (5)
H22C	0.4559	0.7886	0.4795	0.099*	0.688 (5)
O6B	0.5332 (3)	0.6839 (5)	0.4441 (4)	0.0319 (17)	0.312 (5)
C22B	0.5107 (6)	0.7879 (10)	0.5178 (7)	0.033 (2)	0.312 (5)
H22D	0.4482	0.8054	0.5088	0.050*	0.312 (5)
H22E	0.5417	0.8774	0.5083	0.050*	0.312 (5)
H22F	0.5271	0.7513	0.5862	0.050*	0.312 (5)
C21A	0.4969 (5)	1.0087 (11)	0.2733 (6)	0.0490 (17)	0.672 (15)
H21A	0.4501	1.0716	0.2903	0.074*	0.672 (15)
H21B	0.4749	0.9430	0.2193	0.074*	0.672 (15)
H21C	0.5439	1.0664	0.2504	0.074*	0.672 (15)
O7A	0.5288 (3)	0.9287 (5)	0.3606 (5)	0.0431 (15)	0.672 (15)
C21B	0.4989 (11)	1.0049 (18)	0.2305 (13)	0.050 (4)	0.328 (15)
H21D	0.4368	1.0183	0.2108	0.075*	0.328 (15)
H21E	0.5278	0.9793	0.1708	0.075*	0.328 (15)
H21F	0.5237	1.0937	0.2597	0.075*	0.328 (15)
O7B	0.5109 (4)	0.8924 (8)	0.3042 (11)	0.039 (3)	0.328 (15)

### Atomic displacement parameters (Å^2^)

**Table:**

	*U*^11^	*U*^22^	*U*^33^	*U*^12^	*U*^13^	*U*^23^
N1	0.0349 (10)	0.0345 (10)	0.0347 (10)	−0.0060 (8)	0.0082 (8)	−0.0070 (8)
O1	0.0367 (9)	0.0398 (9)	0.0405 (9)	−0.0031 (7)	0.0103 (7)	0.0054 (7)
O2	0.0446 (10)	0.0406 (9)	0.0373 (9)	0.0050 (7)	0.0108 (7)	0.0095 (7)
O3	0.0266 (8)	0.0546 (10)	0.0311 (8)	0.0044 (7)	0.0038 (6)	−0.0008 (7)
O4	0.0374 (9)	0.0410 (9)	0.0244 (8)	0.0063 (7)	0.0044 (6)	−0.0009 (7)
O5	0.124 (2)	0.0637 (14)	0.0779 (15)	0.0484 (14)	0.0743 (15)	0.0347 (12)
C1	0.0502 (14)	0.0356 (13)	0.0443 (14)	0.0007 (11)	0.0134 (11)	0.0089 (11)
C2	0.0410 (12)	0.0273 (11)	0.0304 (11)	0.0100 (9)	0.0103 (9)	0.0017 (9)
C3	0.0299 (11)	0.0259 (10)	0.0369 (12)	0.0035 (8)	0.0092 (9)	−0.0031 (9)
C4	0.0296 (11)	0.0293 (11)	0.0279 (10)	0.0043 (8)	0.0054 (8)	−0.0046 (8)
C5	0.0284 (11)	0.0284 (10)	0.0293 (11)	0.0052 (8)	0.0067 (8)	−0.0053 (9)
C6	0.0267 (10)	0.0311 (11)	0.0309 (11)	0.0031 (8)	0.0064 (8)	−0.0033 (9)
C7	0.0352 (13)	0.0444 (14)	0.0444 (14)	−0.0069 (10)	0.0058 (10)	−0.0143 (11)
C8	0.0288 (12)	0.0584 (16)	0.0362 (13)	0.0021 (11)	0.0018 (9)	−0.0088 (11)
C9	0.0305 (11)	0.0356 (12)	0.0315 (11)	0.0082 (9)	0.0065 (9)	−0.0059 (9)
C10	0.0337 (12)	0.0395 (13)	0.0299 (11)	0.0127 (10)	0.0037 (9)	−0.0016 (9)
C11A	0.0348 (14)	0.113 (3)	0.0433 (15)	0.0206 (16)	0.0076 (11)	0.0161 (17)
C13	0.0259 (10)	0.0338 (11)	0.0244 (10)	−0.0008 (8)	0.0046 (8)	−0.0020 (8)
C14	0.0556 (16)	0.0397 (13)	0.0443 (14)	0.0146 (12)	0.0262 (12)	0.0046 (11)
C15	0.0360 (12)	0.0314 (12)	0.0492 (14)	0.0029 (9)	0.0182 (10)	0.0049 (10)
C16	0.0508 (17)	0.0533 (17)	0.105 (3)	0.0212 (14)	0.0502 (17)	0.0351 (17)
C17	0.0291 (12)	0.0369 (13)	0.090 (2)	0.0080 (10)	0.0183 (13)	0.0169 (14)
C18	0.0405 (13)	0.0312 (11)	0.0342 (12)	0.0024 (10)	−0.0038 (10)	−0.0041 (9)
C19	0.0423 (13)	0.0368 (12)	0.0265 (11)	0.0052 (10)	0.0091 (9)	−0.0039 (9)
C20	0.0249 (10)	0.0290 (11)	0.0258 (10)	−0.0035 (8)	0.0020 (8)	−0.0081 (8)
C12	0.0565 (16)	0.0399 (14)	0.0502 (15)	−0.0148 (12)	0.0127 (12)	−0.0041 (11)
O6A	0.0358 (16)	0.076 (2)	0.0288 (14)	0.0116 (13)	0.0118 (10)	0.0064 (12)
C22A	0.040 (3)	0.114 (5)	0.046 (3)	−0.010 (3)	0.013 (2)	0.017 (3)
O6B	0.027 (3)	0.031 (3)	0.042 (3)	−0.0057 (19)	0.019 (2)	−0.004 (2)
C22B	0.027 (5)	0.038 (5)	0.035 (4)	0.003 (3)	0.007 (3)	−0.002 (4)
C21A	0.037 (2)	0.059 (3)	0.053 (4)	0.012 (2)	0.014 (3)	0.028 (4)
O7A	0.0393 (17)	0.055 (2)	0.037 (3)	0.0203 (15)	0.0117 (17)	0.014 (2)
C21B	0.041 (5)	0.040 (5)	0.072 (10)	0.017 (4)	0.017 (7)	0.033 (8)
O7B	0.031 (3)	0.041 (4)	0.044 (7)	0.008 (2)	0.004 (3)	0.009 (3)

### Geometric parameters (Å, °)

**Table:**

N1—C7	1.465 (3)	C13—H13A	1.0000
N1—C12	1.466 (3)	C14—C15	1.472 (4)
N1—C6	1.469 (3)	C15—C16	1.381 (3)
O1—C3	1.382 (3)	C15—C20	1.382 (3)
O1—C1	1.429 (3)	C16—C17	1.406 (4)
O2—C2	1.379 (3)	C16—O6B	1.425 (5)
O2—C1	1.423 (3)	C16—O6A	1.455 (4)
O3—C4	1.370 (3)	C17—C18	1.391 (4)
O3—C11A	1.400 (3)	C17—O7A	1.392 (4)
O4—C14	1.350 (3)	C17—O7B	1.457 (7)
O4—C13	1.451 (2)	C18—C19	1.392 (3)
O5—C14	1.199 (3)	C18—H18A	0.9500
C1—H1A	0.9900	C19—C20	1.373 (3)
C1—H1B	0.9900	C19—H19A	0.9500
C2—C10	1.366 (3)	C12—H12A	0.9800
C2—C3	1.380 (3)	C12—H12B	0.9800
C3—C4	1.378 (3)	C12—H12C	0.9800
C4—C5	1.413 (3)	O6A—C22A	1.382 (6)
C5—C9	1.398 (3)	C22A—H22A	0.9800
C5—C6	1.517 (3)	C22A—H22B	0.9800
C6—C13	1.546 (3)	C22A—H22C	0.9800
C6—H6A	1.0000	O6B—C22B	1.447 (10)
C7—C8	1.508 (4)	C22B—H22D	0.9800
C7—H7A	0.9900	C22B—H22E	0.9800
C7—H7B	0.9900	C22B—H22F	0.9800
C8—C9	1.500 (3)	C21A—O7A	1.426 (7)
C8—H8A	0.9900	C21A—H21A	0.9800
C8—H8B	0.9900	C21A—H21B	0.9800
C9—C10	1.394 (3)	C21A—H21C	0.9800
C10—H10A	0.9500	C21B—O7B	1.436 (14)
C11A—H11A	0.9800	C21B—H21D	0.9800
C11A—H11B	0.9800	C21B—H21E	0.9800
C11A—H11C	0.9800	C21B—H21F	0.9800
C13—C20	1.496 (3)		
			
C7—N1—C12	109.51 (19)	C6—C13—H13A	109.5
C7—N1—C6	114.66 (17)	O5—C14—O4	120.3 (2)
C12—N1—C6	111.77 (19)	O5—C14—C15	132.0 (2)
C3—O1—C1	104.81 (18)	O4—C14—C15	107.71 (19)
C2—O2—C1	105.30 (18)	C16—C15—C20	122.7 (2)
C4—O3—C11A	118.17 (18)	C16—C15—C14	128.8 (2)
C14—O4—C13	111.50 (17)	C20—C15—C14	108.4 (2)
O2—C1—O1	108.11 (18)	C15—C16—C17	117.2 (2)
O2—C1—H1A	110.1	C15—C16—O6B	121.9 (3)
O1—C1—H1A	110.1	C17—C16—O6B	105.6 (3)
O2—C1—H1B	110.1	C15—C16—O6A	116.8 (3)
O1—C1—H1B	110.1	C17—C16—O6A	124.3 (2)
H1A—C1—H1B	108.4	C18—C17—O7A	127.4 (3)
C10—C2—O2	127.6 (2)	C18—C17—C16	120.3 (2)
C10—C2—C3	122.8 (2)	O7A—C17—C16	111.4 (3)
O2—C2—C3	109.5 (2)	C18—C17—O7B	108.4 (5)
C4—C3—C2	121.3 (2)	C16—C17—O7B	127.2 (4)
C4—C3—O1	128.8 (2)	C17—C18—C19	120.6 (2)
C2—C3—O1	109.82 (19)	C17—C18—H18A	119.7
O3—C4—C3	124.4 (2)	C19—C18—H18A	119.7
O3—C4—C5	118.44 (19)	C20—C19—C18	119.3 (2)
C3—C4—C5	117.1 (2)	C20—C19—H19A	120.4
C9—C5—C4	120.45 (19)	C18—C19—H19A	120.4
C9—C5—C6	121.71 (19)	C19—C20—C15	119.8 (2)
C4—C5—C6	117.82 (19)	C19—C20—C13	131.89 (18)
N1—C6—C5	115.49 (18)	C15—C20—C13	108.27 (19)
N1—C6—C13	109.24 (16)	N1—C12—H12A	109.5
C5—C6—C13	107.95 (17)	N1—C12—H12B	109.5
N1—C6—H6A	108.0	H12A—C12—H12B	109.5
C5—C6—H6A	108.0	N1—C12—H12C	109.5
C13—C6—H6A	108.0	H12A—C12—H12C	109.5
N1—C7—C8	110.9 (2)	H12B—C12—H12C	109.5
N1—C7—H7A	109.5	C22A—O6A—C16	112.2 (3)
C8—C7—H7A	109.5	O6A—C22A—H22A	109.5
N1—C7—H7B	109.5	O6A—C22A—H22B	109.5
C8—C7—H7B	109.5	H22A—C22A—H22B	109.5
H7A—C7—H7B	108.1	O6A—C22A—H22C	109.5
C9—C8—C7	109.8 (2)	H22A—C22A—H22C	109.5
C9—C8—H8A	109.7	H22B—C22A—H22C	109.5
C7—C8—H8A	109.7	C16—O6B—C22B	99.8 (5)
C9—C8—H8B	109.7	O6B—C22B—H22D	109.5
C7—C8—H8B	109.7	O6B—C22B—H22E	109.5
H8A—C8—H8B	108.2	H22D—C22B—H22E	109.5
C10—C9—C5	121.2 (2)	O6B—C22B—H22F	109.5
C10—C9—C8	120.8 (2)	H22D—C22B—H22F	109.5
C5—C9—C8	118.0 (2)	H22E—C22B—H22F	109.5
C2—C10—C9	117.1 (2)	O7A—C21A—H21A	109.5
C2—C10—H10A	121.5	O7A—C21A—H21B	109.5
C9—C10—H10A	121.5	H21A—C21A—H21B	109.5
O3—C11A—H11A	109.5	O7A—C21A—H21C	109.5
O3—C11A—H11B	109.5	H21A—C21A—H21C	109.5
H11A—C11A—H11B	109.5	H21B—C21A—H21C	109.5
O3—C11A—H11C	109.5	C17—O7A—C21A	112.6 (4)
H11A—C11A—H11C	109.5	O7B—C21B—H21D	109.5
H11B—C11A—H11C	109.5	O7B—C21B—H21E	109.5
O4—C13—C20	103.85 (15)	H21D—C21B—H21E	109.5
O4—C13—C6	108.49 (17)	O7B—C21B—H21F	109.5
C20—C13—C6	115.78 (17)	H21D—C21B—H21F	109.5
O4—C13—H13A	109.5	H21E—C21B—H21F	109.5
C20—C13—H13A	109.5	C21B—O7B—C17	123.3 (8)

### Hydrogen-bond geometry (Å, °)

**Table:**

*D*—H···*A*	*D*—H	H···*A*	*D*···*A*	*D*—H···*A*
C6—H6A···O1^i^	1.00	2.54	3.533 (3)	172
C13—H13A···O2^ii^	1.00	2.44	3.317 (3)	146
C18—H18A···O5^iii^	0.95	2.34	3.120 (3)	140

Symmetry codes: (i) −*x*+2, *y*−1/2, −*z*+1/2; (ii) *x*, −*y*+3/2, *z*+1/2; (iii) *x*, −*y*+3/2, *z*−1/2.

## References

[bb1] Anderson, J., Ting, A., Boozer, S., Brunden, K., Crumrine, C., Danzig, J., Dent, T., Faga, L., Harrington, J., Hodrick, W., Murphy, S., Pawlowski, G., Perry, R., Raber, A., Rundlett, S., Stricker-Krongrad, A., Wang, J. & Bennani, Y. (2005). *J. Med. Chem.* **48**, 7096–7098. 10.1021/jm050674q 16279766

[bb2] Aneja, R., Dhiman, N., Idnani, J., Awasthi, A., Arora, S., Chandra, R. & Joshi, H. (2007). *Cancer Chemother. Pharm.* **60**, 831–839. 10.1007/s00280-007-0430-y 17285314

[bb3] Aneja, R., Vangapandu, S. & Joshi, H. (2006). *Bioorg. Med. Chem.* **14**, 8352–8358. 10.1016/j.bmc.2006.09.012 17008104

[bb4] Bergmann, M. & Stolzer, H. (1956). *Wien. Med. Wochenschr.* **106**, 232–233. 13325483

[bb5] Bruker (2007). *SMART*, *SAINT* and *SADABS* Bruker AXS Inc., Madison, Wisconsin, USA.

[bb6] Dahlstrom, B., Mellstrand, T., Lofdahl, C. & Johannsson, M. (1982). *Eur. J. Clin. Pharmacol.* **22**, 535–539. 10.1007/BF00609627 7128665

[bb7] Mahmoudian, M. & Rahimi-Moghaddam, P. (2009). *Recent Patents on Anti-Cancer Drug Discovery*, **4**, 92–97 10.2174/157489209787002524 19149691

[bb8] Seetharaman, J. & Rajan, S. (1995). *Z. Kristallogr.* **210**, 111–113.

[bb9] Sheldrick, G. M. (2008). *Acta Cryst.* A**64**, 112–122. 10.1107/S0108767307043930 18156677

